# A Reassessment of Carbon Content in Tropical Trees

**DOI:** 10.1371/journal.pone.0023533

**Published:** 2011-08-17

**Authors:** Adam R. Martin, Sean C. Thomas

**Affiliations:** Faculty of Forestry, University of Toronto, Earth Sciences Building, Toronto, Ontario, Canada; Centre National de la Recherche Scientifique, France

## Abstract

Accurate knowledge of carbon (C) content in live wood is essential for quantifying tropical forest C stocks, yet generic assumptions (such as biomass consisting of 50% carbon on a weight/weight basis) remain widely used despite being supported by little chemical analysis. Empirical data from stem cores of 59 Panamanian rainforest tree species demonstrate that wood C content is highly variable among co-occurring species, with an average (47.4±2.51% S.D.) significantly lower than widely assumed values. Prior published values have neglected to account for volatile C content of tropical woods. By comparing freeze- and oven-dried wood samples, we show that volatile C is non-negligible, and excluding the volatile fraction underestimates wood C content by 2.48±1.28% (S.D.) on average. Wood C content varied substantially among species (from 41.9–51.6%), but was neither strongly phylogenetically conserved, nor correlated to ecological (i.e. wood density, maximum tree height) or demographic traits (i.e. relative growth rate, mortality rate). Overall, assuming generic C fractions in tropical wood overestimates forest C stocks by ∼3.3–5.3%, a non-trivial margin of error leading to overestimates of 4.1–6.8 Mg C ha^−1^ in a 50-ha forest dynamics plot on Barro Colorado Island, Panama. In addition to addressing other sources of error in tropical forest C accounting, such as uncertainties in allometric models and belowground biomass, compilation and use of species-specific C fractions for tropical tree species would substantially improve both local and global estimates of terrestrial C stocks and fluxes.

## Introduction

Globally, tropical forests constitute a disproportionately large carbon (C) pool, containing roughly 40–50% of all C in terrestrial biomass, despite covering only 7–10% of land area [1]. Moreover, C sources and sinks in tropical forests are highly dynamic even at later stages of forest development. Pan-tropically, old-growth forests have been observed to accumulate C at rates of 0.24–0.63 Mg C ha^−1^ yr^−1^, values which contribute to an estimated net sink of ∼1.3 Pg C yr^−1^ in tropical forests world-wide [1], [2], [3]. At the same time, contributions from tropical forests to increased atmospheric CO_2_ levels from deforestation and degradation account for roughly 12% of total anthropogenic greenhouse gas emissions [4] and dominate national CO_2_ emission profiles in many developing countries such as Brazil and Indonesia [4].

Policy mechanisms, such as Reduced Emissions from Deforestation and Degradation (REDD+), have garnered widespread attention and optimism as a means to slow C emissions from tropical deforestation. Recent studies [5], [6] and commissioned reviews [7] have begun to confirm the economic and ecological viability of such initiatives, particularly in regions or communities with large expanses of primary or secondary tropical forests. However, basic uncertainties exist in our ability to quantify forest C pools and fluxes at the level of accuracy necessary to conduct the highest level, or “Tier 3”, forest C accounting [8]. For instance, although advances have been made in our ability to quantify above-ground biomass (AGB) from forest inventories (e.g. [9], [10], [11]) or remotely sensed data (e.g. [12]), relatively little attention has been given to accurately converting tropical AGB into standing C stocks. This latter oversight has explicit implications for Tier 3 forest C accounting, where IPCC protocols suggest a “specific carbon fraction…should also be incorporated” when estimating C stocks and fluxes in above-ground biomass [8].

Currently, nearly all estimates of tropical forest C pools and fluxes assume all tissues (i.e. wood, leaves, roots) consist of 50% carbon on a dry mass basis (e.g. [1], [2], [13], [14], [15]). Although the Intergovernmental Panel on Climate Change [8] and a few select studies (see [5], [10], [12]) use alternative biomass-carbon conversion factors, data for tropical trees remains scant, and assumptions are generally based on limited chemical analyses that are available. For example, the IPCC [8] biomass-carbon conversion factor for “Tropical and Subtropical Wood” (49%) is based on chemical analysis of a small number (*N* = 3) of pooled samples, each consisting of tissue taken from 5 individual trees, from an undefined set of 15 Amazonian tree species [16]. Similarly, IPCC (2006) conversion factors for woody tissues from tropical and subtropical trees <10 cm and ≥10 cm DBH (46 and 49% respectively) are also based on a small number of pooled samples (*N* = 5 for both conversion factors), each consisting of tissue taken from 15 individual trees, from an undefined set of Mexican rainforest species [17].

In highly diverse tropical forests, overlooking species-specific wood C content reduces the importance of floristic composition as a potential driver of forest C dynamics, and may produce biases in tropical forest C inventories. Generally, woody tissues in trees ≥1 cm DBH comprise the largest fraction (∼95%) of biomass in tropical forests [5], [10], [11], [13], [17]. Yet of all wood functional traits (*sensu* [18]), only wood density (WD) has been explicitly evaluated with regard to tropical forest biomass and C pools to date [9], and very little species-specific wood C content data is available from tropical trees.

Currently there exist only five published accounts of species-specific wood C content for tropical tree species [16], [17], [19], [20], [21]. Of these, only Elias and Potvin [19] provide data for >5 species (32 Panamanian rainforest species). This study also tested relationships between wood C and species' functional traits, reporting a strong relationship between WD and C (*r* ^2^ = 0.86, see Fig. 4 in [19]). This result suggests that 1) WD is a suitable proxy for wood C content, and 2) wood C represents an important axis of life-history variation amongst tropical tree species, similar to that represented by WD [22], [23], [24]. This particular analysis, however, was conducted on a small subset of species (*N* = 9), leaving large uncertainties regarding the generality of these results. To date larger datasets from tropical tree species have not been available to test for functional correlates of wood C content.

In addition, there is an absence of studies on C in tropical woods that account for the volatile carbon fraction, a suite of low-molecular weight “secondary” compounds (e.g. low molecular weight phenolics, terpenoids, aldehydes, etc.) persistent in woody tissues but lost when heated. Recent studies that have freeze-dried fresh temperate tree wood samples, suggest that overlooking the volatile fraction underestimates total wood C content by 1.6–3.5% [25], [26]. However, these studies, despite pointing out the importance of volatile carbon, have not actually derived conversion factors to estimate total live wood C from biomass. Since species-specific biomass estimates are by convention based on oven-dried mass (see [27]), the C content of freeze-dried samples do not accurately apply to oven-dried biomass. Specifically, elemental analysis of freeze-dried wood measures the total C content on a mass/mass basis, such that
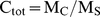
(1)Where C_tot_ is the C content in freeze-dried tissue (free of water), M_C_ is the mass of C in a (freeze-dried) sample, and M_s_ is the total mass of a given sample. This total carbon content of live woody tissues differs from the C conversion factor (C_conv_) applicable to oven-dried AGB:
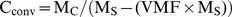
(2)Where VMF is the volatile mass fraction, or mass loss from volatiles attributable to heating samples.

In this study, we sought to redress the lack of accurate C conversion factors in tropical trees, by analyzing the carbon content in woody tissues collected from 59 Panamanian rainforest tree species, the largest dataset from tropical trees to date. This dataset was used to address several questions from an applied forest C accounting perspective and a functional biology perspective: (1) To what extent does wood C content vary among tropical tree species? (2) Is the volatile carbon fraction (C_vol_) an important consideration in tropical forest C accounting? (3) Is wood C content similar among closely related tree taxa (or alternatively, are genus- or family-level C_conv_ values appropriate when species-level information is unavailable)? and (4) Are there strong functional correlates and/or proxy measures of wood C content in tropical trees?

## Materials and Methods

### Ethics Statement

Data and samples used in this research were collected under a Terrestrial Research Permit granted by Panama's National Authority for the Environment (Autoridad Nacional del Ambiente, ANAM), and an “Export Permit for Terrestrial Species (granted by ANAM and Panama's Ministerio de Desarrollo Agropecuario). All permitting applications were facilitated by Helene Muller-Landau at the Smithsonian Tropical Research Institute, Panama.

### Sample Collection and Chemical Analysis

Wood samples were collected in August 2008 at the Pipeline Road site in Soberania National Park (SNP), a lowland tropical moist forest located in central Panama (9°10′N, 75°45′W). Forests in SNP are second-growth, semi-deciduous lowland moist forests with a canopy height of ∼20–40 m, and experience a tropical monsoon climate under the Koppen system of climatic classification [28]. Average rainfall at SNP is ∼2100 mm yr^−1^, and mean monthly temperatures ∼27°C. The forests are seasonal, with a 4-month dry season occurring December through April [28].

A total of 190 wood samples were taken from 59 native tree species across 46 genera, 26 families, and 12 orders (Table S1), with taxonomy following that of the Angiosperm Phylogeny Group 2 (APG2; [29]). We included relatively common species known to grow ≥1 cm DBH. Of our 59 species, 50 are present in nearby (<15 km) 50-ha forest dynamics plot located on Barro Colorado Island (BCI), Panama (9°15′, 79°85′), and in the 2000–2005 census interval these species accounted for 24.3% of all stems and 30.9% of basal area for trees ≥1 cm DBH [30]. Study species were also selected to span a range of life-history strategies from light-demanding pioneer species to shade-tolerant late-successional species, with species-specific growth and mortality rates used as an *a priori* indicator of life-history strategy [23].

For each species, cores were taken from 3–5 individuals ≥10 cm DBH. To avoid biases due to the presence of compression or tension wood, only individual stems with straight growth forms were sampled. Trees with crooked stems, substantial heart-rot, or other forms of stem damage were excluded, and when necessary, cores were taken in directions parallel to slopes, again to avoid compression- and/or tension-wood biases. Cores were taken at breast height (1.3 m above-ground) using a 5.15 mm diameter increment borer, and placed in a freezer within 4 hours of extraction to minimize loss of volatiles.

All wood samples were prepared and analyzed at University of Toronto, Canada. Prior to analysis, the outer edges of the cores were pared away using utility knives to remove oxidized tissue that may have lost volatiles, or may have been contaminated by the surfaces of the core borers. A central portion of the sapwood from each core was then excised, individually pulverized into a homogenous powder using a Wiley Mill (no. 40 mesh), and split for two drying treatments. One half of each sample was placed in a forced-air oven at 110°C for 2 days, the other half was freeze-dried under a vacuum for seven days using a Labconco 8-L freeze drying system (Labconco Co., Kansas City, MO, USA). Dried samples were then analyzed for their carbon content, using an ECS 4010 CN analyzer (Costech Analytical Technologies Inc., Valencia, CA, USA). The analyzer was calibrated between each sample run using an ethylene diamine tetraacetic acid standard.

### Carbon Conversion Factor Calculation

For each sample, we calculated C_conv_ that integrates total C content of freeze-dried wood with the volatile C fraction (C_vol_), expressed relative to oven-dried mass as
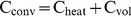
(3)Where C_heat_ is C fraction from elemental analysis of oven-dried samples, and C_vol_ represents the C fraction in volatiles relative to oven-dried mass, such that 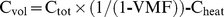
(4)Where C_tot_ is C content in freeze-dried samples, and VMF represents the species' mean mass in volatile compounds lost upon heating. For 29 species, VMF was calculated directly from a subset of samples as
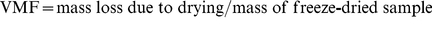
(5)However, due to sample limitations mean VMF was estimated for 30 species as:
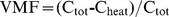
(6)


### Data Analysis – Interspecific Variation in Total- and Volatile Carbon

All statistical analyses were conducted using R v. 2.10.1 (R Foundation for Statistical Computing, Vienna, Austria). We used paired *t*-tests to assess differences between C_conv_ and C_heat_, and two-tailed *t*-tests to compare observed C_conv_ values to 49% and 50% AGB-C conversion factors. Analysis of variance (ANOVA) was used to detect significant differences in C_conv_ and C_vol_ among species, and Spearman's rank correlation test and linear regression was used to evaluate the importance of C_vol_ in driving interspecific variation in C_conv_.

### Data Analysis – Phylogenetic Signal

We examined the phylogenetic signal in C_conv_ and C_vol_ by calculating the *K* statistic [31] using the ‘picante’ R package [32]. The *K* statistic compares a trait distribution across a phylogeny, to the distribution expected under a Brownian motion model of evolution [31], [33]. In this analysis, *K*>1 indicates a trait has a greater phylogenetic signal than expected under Brownian evolution (i.e. a phylogenetically conserved trait), *K*<1 suggests the trait is more randomly distributed across the phylogeny than under a Brownian expectation (i.e. trait convergence across disparate taxa), and *K* = 1 suggests a trait perfectly matches a Brownian model of evolution [31], [33]. We assessed significance of observed *K*-values by randomizing C_conv_ and C_vol_ across the tips of the phylogeny 999 times. Traits are considered significantly conserved if observed *K*-values fell within the 95^th^ percentile of randomized *K*-distributions [33]. It is important to note that the null model (i.e. the randomized trait distribution) used to assess significance of *K*, corresponds to no phylogenetic signal, with *K*_null_<<1 [33]. Phylogenies were created using the software program Phylomatic [34], and were based on APG2 [29]. Unresolved evolutionary relationships were treated as polytomies.

We also used a nested ANOVA (generalized linear mixed model with random effects in the ‘lme4’ R package [35]) to partition variance in C_conv_ and C_vol_ among four nested taxonomic levels (species within genus within family within order). In this analysis, the cumulative variation explained as one moves from higher to lower taxonomic levels (i.e. from order to family to genus to species) is interpreted as the *intra*-*class correlation* in C_conv_ and C_vol_, or “the correlation expected between any two data points selected at random from the same (taxonomic) group” such as two species from the same genus, or two genera from the same family [36].

### Data Analysis – Ecological Correlates

Ecological correlates of wood C content (C_conv_) examined in this study were relative growth rate (RGR), mortality rate (M), maximum tree height (H_max_), and WD. RGR and M data were taken from Condit et al. [37], and are expressed as the percentages calculated for individuals ≥10 cm DBH at the BCI forest dynamics plot. Published WD figures were available for 25 study species [38], and WD for the remaining 34 species were provided by S.J. Wright (unpublished data; Table S1). Published and unpublished WD values were calculated using the identical methodologies (see [38]), and were in nearly all cases derived from the same trees cored for wood C analysis in this study. H_max_ data was taken from two published sources [38], [39], and unpublished data provided by R. Condit (Table S1). Species' H_max_ from unpublished data was calculated as the mean height of the three largest trees by DBH in the dataset [38]. For a small set of species (*N* = 23), information was also available for the 95^th^ percentile of the fastest growing individuals on BCI (RGR_95_), and mortality of the 25th percentile of slowest growing individuals on BCI (M_25_). RGR_95_ and M_25_ were taken from Wright et al. [38] and are expressed in cm cm^−1^ yr^−1^, and % 5 yr^−1^, respectively. Prior to analysis RGR, RGR_95_, M, and M_25_ were log-transformed to meet assumptions of normality.

We used step-wise linear regression analysis with species-level mean C_conv_ as the dependent variable, to identify functional correlates of C_conv_. Models were compared using Akaike's information criteria (AIC), with the lowest AIC indicating the most parsimonious explanatory model. Significance of independent variables in the AIC-selected model was determined using multiple regression. Step-wise regression and AIC-model comparisons were conducted on the subset of species (*N* = 32) for which data on RGR, M, WD, and H_max_ were available. Linear regression was used to test for relationships between C_conv_ and RGR_95_ and M_25_ separately, due to sample size limitations.

## Results

### Interspecific Variation in Total and Volatile Carbon

**Figure 1:**
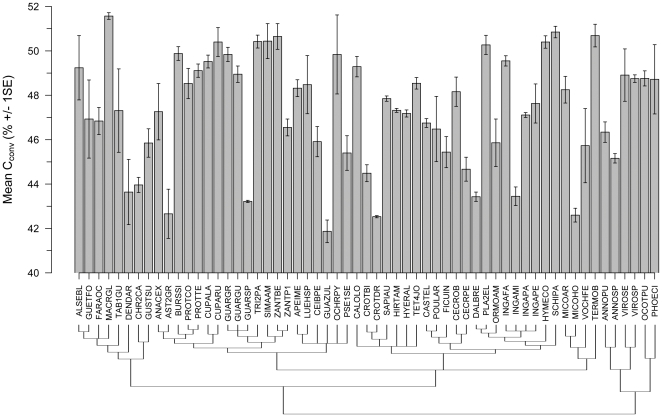
Mean C conversion factors (C_conv_) across 59 Panamanian rainforest tree species. C_conv_ averaged 47.35±0.33% (S.E.) ranging from 41.87±0.51% (S.E.) in *Guazuma ulmifolia* (GUAZUL), to 51.57±0.15% (S.E.) in *Macrocnemum roseum* (MACRGL). C_conv_ differed significantly among species (*N* = 193, *F*_58,131_ = 6.55, *P*<0.0001), and is not phylogenetically conserved (*K* = 0.186, *P* = 0.803). Error bars represent ±1 standard error of the mean, and the tree represents phylogenetic relationships among species as per APG2. Species codes are defined in Table S1.

Carbon conversion factors (C_conv_) varied significantly among species (*F*_58,131_ = 6.55, *P*<0.0001; Fig. 1), averaging 47.35±2.51% (S.D.) and ranging between 41.87±0.89% (S.D.) (*Guazuma ulmifolia*) and 51.57±0.29% (S.D.) (*Macrocnemum roseum*; Fig. 1). Average C_heat_ also differed significantly among species (*F*_58,131_ = 5.90, *P*<0.0001); average C_heat_ (44.99±1.49% S.D.) was significantly lower than C_conv_ (one-sided paired *t*-test, *t*_58_ = 12.58, *P*<0.0001). Although our minimum observed value for C_heat_ samples was similar to C_conv_ samples (41.89±0.45% S.D. in *Miconia hondurensis*), the oven-drying treatment reduced the maximum observed C_heat_ value to 48.19±0.22% (S.D.) (*Macrocnemum roseum*).

Our observed mean C_conv_ were significantly lower than widely assumed AGB-C conversion factors. As compared to the IPCC [8] for “Tropical and Subtropical Wood” (49%) our mean C_conv_ was 1.65% lower on average (two-tailed *t*-test, *t*_58_ = −5.05, *P*<0.0001), while our observed values were 2.65% lower on average than a 50% conversion factor (two-tailed *t*-test, *t*_58_ = −8.11, *P*<0.0001).

**Figure 2:**
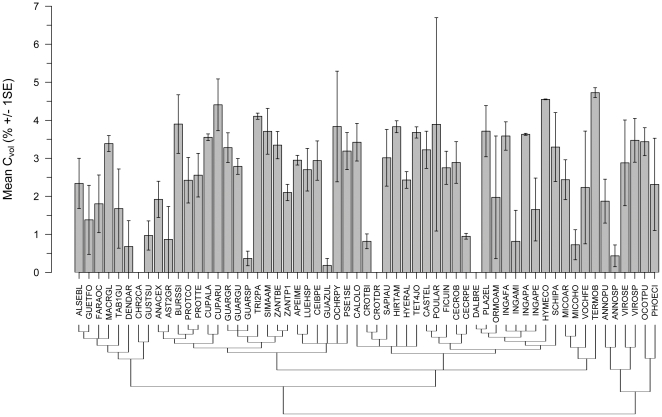
Mean volatile carbon fraction (C_vol_) in woody tissues of 59 Panamanian rainforest tree species. C_vol_ averaged 2.48±0.17% (S.E.) among species, ranging between non-detectable in three species, to 4.73±0.13 (S.E.) in *Terminalia oblonga* (TERMOB). C_vol_ differed significantly among species (*N* = 190, *F*_58,131_ = 2.83, *P*<0.0001), is significantly greater than 0 (*t*_58_ = 14.84, *P*<0.0001), but not phylogenetically conserved (*K* = 0.206, *P* = 0.583). Error bars represent ±1 standard error of the mean, and the tree represents phylogenetic relationships among species as per APG2. Species codes are defined in Table S1.

**Figure 3:**
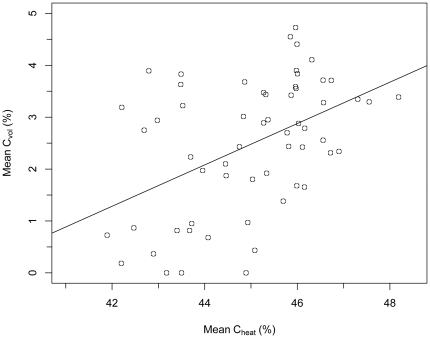
Volatile carbon content (C_vol_) as a function of oven-dried wood C content (C_heat_). Trend-line represents a linear regression model where C_vol_ = (C_heat_ * 0.40)−15.46 (*N* = 59 species, adj. *r* ^2^ = 0.20, *P* = 0.0002).

In tropical hardwoods, C_vol_ in woody tissues was non-negligible. Wood C differed significantly with drying treatment (one-tailed paired *t*-test, *t*_58_ = 12.58, *P*<0.0001), and corresponding estimates of C_vol_ pooled across all species were significantly greater than 0 (one-tailed *t*-test, *t*_58_ = 14.84, *P*<0.0001). Additionally, the C_vol_ differed significantly among species (*F*_58,131_ = 2.83, *P*<0.0001; Fig. 2), averaging 2.48±1.28% (S.D.), and ranging from non-detectable (0%) in three species (*Croton draco*, *Chrysophyllum cainito*, *Dalbergia retusa*) to 4.73% in *Terminalia oblonga* (Fig. 2). Species with a larger C_vol_ also had greater total wood C as evidenced by a strong (though imperfect) positive C_conv_- C_vol_ rank correlation (Spearman's ρ = 0.8, *P*<0.0001, *N* = 59). We also observed a significant positive correlation between C_vol_ and C_heat_ (adjusted *r* ^2^ = 0.20, *P* = 0.0002, Fig. 3), suggesting that in absolute terms C_vol_ tended to be higher in species with greater “structural” C content.

### Phylogenetic Variation in Total- and Volatile Carbon

Overall, we found no evidence for phylogenetic conservatism in wood C content (Fig. 1, Table 1). Observed *K* for C_conv_ (*K* = 0.186) suggests this trait is more randomly distributed across the phylogeny than would be expected under Brownian trait evolution. Additionally, observed *K*-value for C_conv_ did not fall in the upper 95^th^ percentile of the randomized *K*-distribution (*P* = 0.792). Although some congeneric species showed similarity in C_conv_ (e.g. <1% difference among *Cupania*, *Protium*, and *Virola* species), the lack of phylogenetic signal in C_conv_ is driven by large divergences in other genera. For instance, congeneric species in *Cecropia*, *Guarea*, *Inga*, *Miconia*, and *Zanthoxylum* differed by ≥3.0% in C_conv_.

**Table 1:** Explained variation, and cumulative explained variation/intra-class correlations in C_conv_ (*N* = 193) and C_vol_ (*N* = 190) at 4 nested taxonomic levels.

	C_conv_		C_vol_	
Taxonomic level	Variance explained (%)	Intra-class correlation	Variance explained (%)	Intra-class correlation
Order	0.92	0.92	0.00	0.00
Family	0.00	0.92	0.00	0.00
Genus	0.00	0.92	9.45	9.45
Species	62.78	63.70	26.96	36.40
Total explained	63.70	NA	36.40	NA
Unexplained	36.30	NA	63.60	NA

Nested ANOVA provided additional support for this trend. Although taxonomic information alone explained a total of 63.7% variation in C_conv_ among samples (*N* = 190), the large majority of variation was explained at the species level. Species identity accounted for 62.8% variance in C_conv_, or 98.6% of the total variance explained by taxonomy (Table 1). Interestingly, genus- and family-level identity explained 0% of the variation in C_conv_, indicating that congeneric or co-family pairs are not more similar in wood C content than a randomly selected set of species. Order-level taxonomic identity accounted for 0.91% of the total variation, or 0.01% of the total explained variation.

Similarly, C_vol_ was not conserved across the phylogeny (*K* = 0.206, *P* = 0.583, Fig. 2): a result supported by nested ANOVA. Taxonomic information explained 36.4% of the total variation in C_vol_ (*N* = 190 samples), with species-and genus-level terms explaining the entirety of this variance (Table 1). Species terms explained 26.96% of the variation in C_vol_ (or 74.1% of the explained variance), while genus identity explained 9.45% of the variation in C_vol_ (or 25.9% of the variance explained by taxonomy). Family and order identity accounted for 0% of the variation in C_vol_ (Table 1). In total, 63.6% of the variation in C_vol_ remained unaccounted for by taxonomic information.

### Ecological Correlates of Wood Carbon

**Figure 4:**
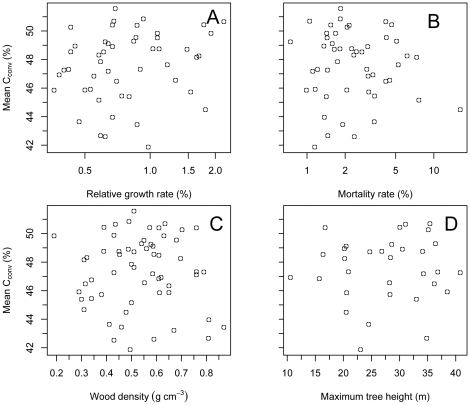
Carbon conversion factors (C_conv_) as a function of four ecological correlates. All ecological and/or demographic species' traits tested were unrelated to C_conv_ (4A: log-RGR, *N* = 49 species, *P* = 0.184. 4B: log-M, *N* = 49 species, *P* = 0.674. 4C: WD, *N* = 59 species, *P* = 0.735. 4D: H_max_, *N* = 32 species, *P* = 0.791).

Step-wise regression indicated linear combinations of two or more species' traits did not explain variation in C_conv_ (*P*≥0.74, adj. *r* ^2^<0 in three multiple regression models where *N* = 32 species); rather, log-RGR alone was the most parsimonious predictor of C_conv_. However, when applied to the entire dataset for which RGR data was available (*N* = 49 species), this relationship was not significant (adj. *r* ^2^ = 0.017, *P* = 0.184: Fig. 4A). Similarly, we found no significant bivariate relationships between C_conv_, and our three other ecological variables across the entire dataset (log-M, *N* = 49 species, *P* = 0.674; WD, *N* = 59 species, *P* = 0.735; H_max_, *N* = 32 species, *P* = 0.791: Fig. 4B-D, respectively). Our dataset did not detect a strong WD-C relationship (adj. *r* ^2^ = −0.016, *P* = 0.735, Fig. 4C), and C_conv_ was also unrelated to RGR_95_ (adj. *r* ^2^<0, *P* = 0.425) and M_25_ (adj. *r* ^2^<0, *P* = 0.324; data not shown).

## Discussion

We found that live wood C content (expressed as a percentage of wood dry mass) was highly variable among tropical hardwood species (Fig. 1, Table S1), and on average significantly lower than assumed in the scientific literature. Our study also confirms that the volatile carbon fraction (C_vol_) is an important component of total wood C content in tropical species (Figs. 2 and 3, Table S1), indicating that neglecting this fraction will significantly underestimate total wood C content. Lastly, our study showed that wood C content and C_vol_ are neither phylogenetically conserved (Figs. 1 and 2, Table 1), nor strongly correlated to ecological and/or demographic traits examined (Fig. 4A–D): results thus suggesting that mean C_conv_ values derived from tropical trees, and not higher-taxon C_conv_ values or proxy measures, are most appropriate for use in forest C accounting protocols (e.g. [8]) in the absence of species-specific carbon content information.

### Interspecific variation and the adaptive significance of wood C content

In woody tissues of 59 Panamanian rainforest tree species, interspecific variation in C_vol_ contributed significantly to among-species variation in total wood C content. The range of C_vol_ values was 4.73%, while C_conv_ varied by 9.7% (Fig. 1 and 2). Thus a large component of interspecific variation in wood C among tropical hardwoods is due to differences in the solid-phase chemical constituents of wood, the most abundant of which are cellulose and lignin [40]. Proportions of these compounds are variable in tropical hardwoods, and on a dry mass basis cellulose (including hemicellulose and cellobiose) constitutes ∼65–75% of woody tissues while lignin constitutes ∼20–50% [21], [40], [41]. The C content of these compounds differs greatly, with cellulose containing 40–44% C and lignins 60–72% [21], [40], [42]. Thus cellulose: lignin ratios between ∼2.5–4 likely account for much of the variation in wood C content in tropical hardwoods. This trend is supported by existing data: augmenting our data with published lignin content values [40], [41], C_heat_ from 14 Neotropical tree species (11 from this study, plus 3 approximated from Fig. 1 in [19]) is significantly positively correlated with lignin content on a percent dry mass basis (*P* = 0.008, *r* ^2^ = 0.4074, where C_heat_ = 33.34+(0.29 * %lignin); data not shown). Analyzing the correlation between cellulose: lignin ratios and wood C content for a larger number of tropical species is necessary to confirm the generality of this relationship.

Another likely source of interspecific variation in wood C content is variation in non-structural carbohydrate (NSC) concentrations. NSCs resemble cellulose in terms of C content (∼42%), and comprise ∼5–20% of dry mass in stems of tropical hardwoods [43], [44]. Thus, in general, higher NSC concentrations will tend to reduce wood C content, when expressed as a percentage of dry mass. Within tropical saplings NSCs are generally found in higher concentrations in slow-growing, shade-tolerant tree species [45]. Thus, one might expect wood C will closely track variation in light requirements/demographic rates, with lower total C content observed in slower growing, shade tolerant species due to higher NSC concentrations. However, our data did not support this relationship, as evidenced by a lack of significant relationships between C_conv_ and log-RGR, log-M, or WD (Fig. 4A–C). Additionally, H_max_, a trait representing a species' light capture strategy [46], did not correlate with C_conv_ (Fig. 4D). Our analyses thus suggest that while wood C content varies significantly among tropical tree species, it is unrelated to functional traits examined here. We speculate that the trend toward higher NSCs in shade-tolerant tree species (which would decrease C_conv_) may be offset by an increased lignin-cellulose ratio (which would increase C_conv_). Further analyses of NSC as well as physiological traits associated with C assimilation and storage may provide additional insights.

### Tropical Forest Carbon Accounting

Mean C_conv_ from our 59 study species (47.35%) were greater than median C values from previous tropical studies (∼46% in [19]). The absolute differences in C values between these studies (∼1.35%) is approximately half of the value of our observed mean C_vol_ (2.8%), suggesting that observed differences are mainly due to loss of volatiles on heating of samples. We suggest that for tropical hardwoods in natural forests, a mean biomass-C conversion factor of 47.4% is currently the most reliable, analytically supported value for wood C content. Ideally, a large database similar to that for WD [18], [47] containing species-specific C information is needed to accurately estimate tropical forest C stocks, particularly for common species. Within our study region, addition of two species (*Trichilia tuberculata* (Meliaceae) and *Quararibea asterolepis* (Malvaceae)) would have provided additional C information for 14.1% of total AGB stocks (based on the 2000 census at BCI; [11]). Pantropically, better knowledge of species-specific wood C values would have immediate implications for forest C accounting, with some of the most compelling examples coming from monodominant forests. For instance, in the Eastern Congo Basin, analysis of the C content in just one species (*Gilbertiodendron dewevrei* (Fabaceae)) would resolve ∼60% of C accounting error associated with AGB-C conversion [48].

The bias associated with AGB-C conversion, as indicated by our results, arises from a significant overestimate of forest C stocks due to use of conventional conversion factors. On average, as compared to C_conv_ for our 59 Panamanian species, the 49% conversion used by the IPCC [8] overestimates forest C stocks by 3.3%, while assuming 50% C overestimates forest C stocks by 5.3%. To illustrate the magnitude of this error, we calculated forest C stocks for all live stems ≥1 cm DBH, based on AGB data from four censuses between 1985–2000 at the 50-ha forest dynamics plot on BCI [11]. Across the census intervals (*N* = 4), conversion of AGB to C stocks using 50% and 49% [8] carbon fractions yield 136.8±1.1 and 134.1±1.0 Mg C ha^−1^, respectively. We calculate this forest to hold 129.9±1.0 Mg C ha^−1^ when converting AGB to C with species-specific C_conv_ values, and 47.4% C_conv_ for species not included in our study. Therefore, in the BCI example, assuming a generic C fraction for tropical trees overestimates aboveground C stocks by 4.1–6.8 Mg C ha^−1^. This degree of error will compound substantially at larger spatial scales. In a recent pantropical analysis Lewis et al. [1] estimated tropical forests are globally a net C sink over recent decades, and based on a 50% C fraction sequester C in live AGB at a rate of 0.9 Pg C yr^−1^ (95% CI, 0.5–1.2). Yet when converted using our mean C_conv_ value, this sink is closer to 0.85 Pg C yr^−1^. Although this value falls within their 95% confidence intervals, this represents an easily corrected bias: substituting our mean C_conv_ values, the mean global C accumulation rates and associated confidence intervals presented by Lewis et al. [1] would be reduced by roughly 50 million Mg C yr^−1^.

Our dataset also suggests that deriving wood C fractions for tropical trees by oven-drying wood samples will introduce underestimates in C stocks by 1.9% on average (Fig. 2). Again converting 2000 AGB from BCI [11] using species-specific C_heat_ values, and a C_heat_ mean of 44.99% for species not in our studies, suggests that omitting the C_vol_ underestimates C stocks by 6.6 Mg C ha^−1^, with the largest underestimates coming from common species, and those with higher total wood C content such as *Terminalia oblonga* and *Cupania rufescens* (Figs. 1 and 3). Larger underestimates due to oven-drying in species with higher overall C content would be expected, given the significant positive relationship between structural carbon (i.e. C_heat_) and C_vol_ (Fig. 3). Biologically, the observed positive relationship between structural C and C_vol_ likely owes to common volatile compounds such as coniferyl alcohol that are requisite precursors to lignin [49].

Overall, underestimates in C accounting attributable to C_vol_ omission in tropical trees are comparable to current data from temperate species (e.g. 2% in two North American conifers [26], and 3.5% in one temperate Chinese conifer [25]). However, exact comparisons with existing temperate studies are difficult due to methodological discrepancies: in studies of Chinese [25] and North American [26] species, C_vol_ was calculated as the difference between C_tot_ and C_heat_, inconsistent with Equation 4 here. When standardized, temperate trees would likely show larger C_vol_ than tropical trees, due to high volatile C content found in temperate conifers. Yet for certain tropical forests, if common species possess large C_vol_ values, forest C accounting errors associated with omitting the C_vol_ in tropical forests may be larger than our data suggest.

### Conclusion

Within the larger context of tropical forests C accounting, resolving uncertainties in wood C fractions addresses one of several inaccuracies that remain [50]. For instance, the ability of allometric models to accurately predict tree AGB remains relatively unclear when tree-specific traits (e.g. tree height, WD) are not measured [9], [51], and few allometric models have been parameterized for African forest trees [14]. Also, estimating belowground biomass/C in tropical forests has received surprisingly little attention [50], and for a given site is generally estimated as 24–37% of AGB [5], [8], [52], [53], with a near complete lack of information for C fractions of tropical tree roots. Here we show that stem wood C content is highly variable among co-occurring tropical tree species, variation that has to date been overlooked in scientific studies and carbon inventories. Recalculating Panamanian forest C stocks, and pantropical forest C fluxes using our analytically-derived wood C fractions, we show that use of common generic conversion factors leads to substantial overestimates in forest C inventories: non-trivial errors which have important implications for high-level (Tier 3) forest C accounting [8]. There is thus an urgent need to accumulate C_conv_ data from tropical tree species across a range of tropical forest sites. This is essential both for understanding the functional biology of variation in wood C content in tropical trees, and for deriving accurate estimates of C stocks throughout tropical forests globally.

## Supporting Information

Table S1 **Taxonomy, species code, and C parameters for woody tissues of 59 Panamanian rainforest tree species.** C parameters are expressed as species' mean (weight/oven-dried weight) ± S.E. Species codes correspond to Fig. 1 and 2. Superscripts following taxonomy refer to sources for H_max_ and WD, respectively: ^*^Wright et al. [38], ^†^King et al. [39], ^‡^Wright, S.J. (unpublished data), ^¶^Condit, R. (unpublished data).
(DOC)
