# Supplementary material for: A Reassessment of Carbon Content in Tropical Trees
Source: PLoS One. 2011 Aug 17;6(8):e23533. doi: 10.1371/journal.pone.0023533 (PMC3157388; doi:10.1371/journal.pone.0023533)
Supplement: Table S1 — Taxonomy, species code, and C parameters for woody tissues of 59 Panamanian rainforest tree species. C parameters are expressed as species' mean (weight/oven-dried weight) ± S.E. Species codes correspond to Fig. 1 and 2. Superscripts following taxonomy refer to sources for Hmax and WD, respectively: *Wright et al. [38], †King et al. [39], ‡Wright, S.J. (unpublished data), ¶Condit, R. (unpublished data). (DOC) [file pone.0023533.s001.doc]

**Table S1**.

| Species | Code | C_conv_ | C_heat_ | C_vol_ |
| --- | --- | --- | --- | --- |
| Alseis blackiana (Rubiaceae)^1, 1^ | ALSEBL | 49.24 ± 1.45 | 46.90 ± 0.89 | 2.34 ± 0.66 |
| Anacardium excelsum (Anacardiaceae)^4, 3^ | ANACEX | 47.26 ± 1.27 | 45.34 ± 0.92 | 1.92 ± 0.48 |
| Annona purpurea (Annonaceae)^NA, 3^ | ANNOPU | 46.34 ± 0.47 | 44.46 ± 0.19 | 1.87 ± 0.58 |
| Annona spraguei (Annonaceae)^NA, 3^ | ANNOSP | 45.16 ± 0.21 | 45.08 ± 0.69 | 0.44 ± 0.29 |
| Apeiba membranacea (Tiliaceae)^1, 1^ | APEIME | 48.32 ± 0.38 | 45.37 ± 0.30 | 2.95 ± 0.13 |
| Astronium graveolens (Anacardiaceae)^1, 1^ | AST2GR | 42.66 ± 1.11 | 42.47 ± 0.25 | 0.87 ± 0.87 |
| Bursera simaruba (Burseraceae)^NA, 3^ | BURSSI | 49.88 ± 0.31 | 45.98 ± 0.47 | 3.90 ± 0.77 |
| Calophyllum longifolium (Clusiaceae)^1, 1^ | CALOLO | 49.29 ± 0.46 | 45.87 ± 0.54 | 3.42 ± 0.50 |
| Castilla elastica (Moraceae)^NA, 3^ | CASTEL | 46.75 ± 0.20 | 43.52 ± 0.37 | 3.22 ± 0.49 |
| Cecropia obtusifolia (Cecropiaceae)^NA, 3^ | CECROB | 48.16 ± 0.66 | 45.27 ± 0.28 | 2.89 ± 0.55 |
| Cecropia peltata (Cecropiaceae)^NA, 3^ | CECRPE | 44.67 ± 0.54 | 43.72 ± 0.61 | 0.95 ± 0.08 |
| Ceiba pentandra (Bombacaceae)^4, 3^ | CEIBPE | 45.92 ± 0.68 | 42.96 ± 0.64 | 2.94 ± 0.52 |
| Chrysophyllum cainito (Sapotaceae)^NA, 3^ | CHR2CA | 43.96 ± 0.35 | 44.89 ± 0.05 | Non-detectable |
| Croton billbergianus (Euphorbiaceae)^1, 1^ | CROTBI | 44.49 ± 0.38 | 43.67 ± 0.25 | 0.82 ± 0.20 |
| Croton draco (Euphorbiaceae)^NA, 3^ | CROTDR | 42.53 ± 0.05 | 43.18 ± 0.13 | Non-detectable |
| Cupania latifolia (Sapindaceae)^NA, 3^ | CUPALA | 49.52 ± 0.29 | 45.97 ± 0.36 | 3.55 ± 0.09 |
| Cupania rufescens (Sapindaceae)^NA, 3^ | CUPARU | 50.40 ± 0.65 | 45.99 ± 0.04 | 4.41 ± 0.68 |
| Dalbergia retusa (Fabaceae)^NA, 3^ | DALBRE | 43.45 ± 0.21 | 43.50 ± 0.23 | Non-detectable |
| Dendropanax arboreus (Araliaceae)^1, 1^ | DENDAR | 43.64 ± 1.47 | 44.07 ± 0.92 | 0.68 ± 0.68 |
| Faramea occidentalis (Rubiaceae)^1, 1^ | FARAOC | 46.84 ± 0.61 | 45.03 ± 0.70 | 1.80 ± 0.76 |
| Ficus insipida (Moraceae)^NA, 3^ | FICUIN | 45.44 ± 0.70 | 42.70 ± 0.35 | 2.75 ± 0.44 |
| Guarea grandifolia (Meliaceae)^NA, 3^ | GUARGR | 49.84 ± 0.32 | 46.56 ± 0.10 | 3.28 ± 0.39 |
| Guarea guidonia (Meliaceae)^1, 1^ | GUARGU | 48.95 ± 0.37 | 46.16 ± 0.25 | 2.79 ± 0.21 |
| Guarea 'fuzzy' (Meliaceae)^NA, 3^ | GUARSP | 43.22 ± 0.05 | 42.89 ± 0.21 | 0.37 ± 0.19 |
| Guazuma ulmifolia (Sterculiaceae)^1, 1^ | GUAZUL | 41.87 ± 0.51 | 42.20 ± 0.43 | 0.18 ± 0.18 |
| Guettarda foliacea (Rubiaceae)^1, 1^ | GUETFO | 46.93 ± 1.76 | 45.70 ± 0.84 | 1.38 ± 0.91 |
| Gustavia superba (Lecythidaceae)^1, 1^ | GUSTSU | 45.85 ± 0.64 | 44.93 ± 0.37 | 0.97 ± 0.38 |
| Hirtella americana (Chrysobalanaceae)^2, 3^ | HIRTAM | 47.32 ± 0.09 | 43.49 ± 0.23 | 3.83 ± 0.16 |
| Hieronyma alchorneoides (Euphorbiaceae)^1, 1^ | HYERAL | 47.18 ± 0.17 | 44.75 ± 0.06 | 2.43 ± 0.22 |
| Hymenaea courbaril (Fabaceae)^NA, 3^ | HYMECO | 50.40 ± 0.28 | 45.85 ± 0.29 | 4.55 ± 0.01 |
| Inga laurina (Fabaceae)^NA, 3^ | INGAFA | 49.55 ± 0.23 | 45.96 ± 0.19 | 3.59 ± 0.37 |
| Inga oerstediana (Fabaceae)^NA, 3^ | INGAMI | 43.45 ± 0.42 | 43.40 ± 0.79 | 0.82 ± 0.82 |
| Inga pauciflora (Fabaceae)^NA, 3^ | INGAPA | 47.11 ± 0.11 | 43.48 ± 0.10 | 3.63 ± 0.03 |
| Inga pezizifera (Fabaceae)^NA, 3^ | INGAPE | 47.63 ± 0.88 | 46.15 ± 0.14 | 1.65 ± 0.83 |
| Luehea speciosa (Tiliaceae)^NA, 3^ | LUEHSP | 48.48 ± 1.31 | 45.78 ± 0.84 | 2.7 ± 0.56 |
| Macrocnemum roseum (Rubiaceae)^NA, 3^ | MACRGL | 51.57 ± 0.15 | 48.19 ± 0.11 | 3.39 ± 0.21 |
| Miconia argentea (Melastomataceae)^1, 1^ | MICOAR | 48.25 ± 0.60 | 45.81 ± 0.31 | 2.44 ± 0.52 |
| Miconia hondurensis (Melastomataceae)^NA, 3^ | MICOHO | 42.60 ± 0.31 | 41.89 ± 0.26 | 0.73 ± 0.40 |
| Ochroma pyramidale (Bombacaceae)^NA, 3^ | OCHRPY | 49.84 ± 1.79 | 46.00 ± 0.44 | 3.82 ± 1.46 |
| Ocotea puberula (Lauraceae)^1, 1^ | OCOTPU | 48.76 ± 0.34 | 45.32 ± 0.29 | 3.44 ± 0.37 |
| Ormosia amazonica (Fabaceae)^NA, 3^ | ORMOAM | 45.86 ± 1.07 | 43.96 ± 0.66 | 1.97 ± 1.61 |
| Cinnamomum triplinerve (Lauraceae)^2, 3^ | PHOECI | 48.72 ± 1.56 | 46.72 ± 0.14 | 2.31 ± 1.21 |
| Platypodium elegans (Fabaceae)^1, 1^ | PLA2EL | 50.27 ± 0.43 | 46.55 ± 0.27 | 3.71 ± 0.67 |
| Poulsenia armata (Moraceae)^1, 1^ | POULAR | 46.48 ± 1.47 | 42.79 ± 1.80 | 3.89 ± 2.81 |
| Protium costaricense (Burseraceae)^1, 1^ | PROTCO | 48.53 ± 0.68 | 46.11 ± 0.09 | 2.42 ± 0.60 |
| Protium tenuifolium (Burseraceae)^1, 1^ | PROTTE | 49.12 ± 0.31 | 46.56 ± 0.30 | 2.56 ± 0.58 |
| Pseudobombax septenatum (Bombacaceae)^4, 3^ | PSE1SE | 45.40 ± 0.78 | 42.21 ± 1.23 | 3.19 ± 0.49 |
| Sapium glandulosum (Euphorbiaceae)^NA, 3^ | SAPIAU | 47.85 ± 0.12 | 44.84 ± 0.65 | 3.01 ± 0.75 |
| Schizolobium parahyba (Fabaceae)^NA, 3^ | SCHIPA | 50.85 ± 0.26 | 47.55 ± 0.82 | 3.30 ± 0.91 |
| Simarouba amara (Simaroubaceae)^1, 1^ | SIMAAM | 50.44 ± 0.79 | 46.73 ± 0.20 | 3.71 ± 0.60 |
| Tabebuia guayacan (Bignoniaceae)^1, 1^ | TAB1GU | 47.31 ± 1.88 | 45.99 ± 0.75 | 1.68 ± 1.04 |
| Terminalia oblonga (Combretaceae)^1, 1^ | TERMOB | 50.69 ± 0.51 | 45.96 ± 0.64 | 4.73 ± 0.13 |
| Tetrathylacium johansenii (Flacourtiaceae)^NA, 3^ | TET4JO | 48.54 ± 0.26 | 44.86 ± 0.37 | 3.68 ± 0.15 |
| Trichilia pallida (Meliaceae)^1, 1^ | TRI2PA | 50.42 ± 0.29 | 46.31 ± 0.25 | 4.11 ± 0.08 |
| Virola sebifera (Myristicaceae)^1, 1^ | VIROSE | 48.91 ± 1.18 | 46.03 ± 0.18 | 2.88 ± 1.13 |
| Virola multiflora (Myristicaceae)^2, 3^ | VIROSP | 48.75 ± 0.17 | 45.27 ± 0.55 | 3.47 ± 0.58 |
| Vochysia ferruginea (Vochysiaceae)^4, 3^ | VOCHFE | 45.73 ± 1.67 | 43.69 ± 0.05 | 2.23 ± 1.48 |
| Zanthoxylum ekmanii (Rutaceae)^1, 1^ | ZANTBE | 50.65 ± 0.58 | 47.31 ± 0.27 | 3.35 ± 0.36 |
| Zanthoxylum panamense (Rutaceae)^1, 1^ | ZANTP1 | 46.55 ± 0.39 | 44.45 ± 0.44 | 2.10 ± 0.21 |
